# Peer Review of “Continuous User Experience Monitoring of a Patient-Completed Preoperative Assessment System in the United Kingdom: Cross-sectional Study”

**DOI:** 10.2196/35507

**Published:** 2022-01-06

**Authors:** Andrea N Mahnke

**Affiliations:** 1 Customer Experience Department Marshfield Clinic Health System Marshfield, WI United States; 2 Biomedical & Health Informatics University of Wisconsin-Milwaukee Milwaukee, WI United States

*This is a peer-review report submitted for the paper “Continuous User Experience Monitoring of a Patient-Completed Preoperative Assessment System in the United Kingdom: Cross-sectional Study.”*

## Round 1 Review

### General Comments

This paper [1] highlights important usability evaluations of an assessment system used by patients. These systems are often pushed out to patients without any evaluation of whether patients can use them successfully and if they are satisfying to interact with.

### Specific Comments

#### Minor Comments

1. The staggering of the eHealth usability scale items was a good idea. It would have been most likely unrealistic for patients to be willing to complete the scale including all of the items.
2. Besides further studying what things impacted completion times, it would also be helpful to further explore the impact of a message addressing the length of assessment completion at the start of the assessment.
